# The relationship between out-of-hours admission to icu and patient outcome

**DOI:** 10.1186/2197-425X-3-S1-A961

**Published:** 2015-10-01

**Authors:** A Gupta, P Morgan

**Affiliations:** Surrey and Sussex Hospitals Trust, Intensive Care, Redhill, United Kingdom

## Introduction

At present, there is a paucity of information delineating the relationship between patient outcome and after-hours admissions to ICU. Studies have demonstrated either higher mortality rates or have shown no increased risk with off-hours ICU admissions, even when confounding factors are accounted for [1–3].

## Objectives

To evaluate the relationship between the mortality rates of patients admitted to ICU and their time of their admission.

## Methods

Retrospective cohort study (n = 9092) of patients aged between 17-98 years, admitted to SASH ICU between Dec 1992-Dec 2014 were categorised into out-of-hours (17:00-08:00) or in-hours (08:00-17:00) admission. Mortality rates in each group were calculated. Elective surgical admissions were excluded.

Statistical Analysis:

Categorical data, analysed using Fisher's, Chi-Square and 2 × 2 contingency tables.

## Results

Of the 9092 patients, 5765 were admitted out-of-hours and 3327 were admitted in-hours.

There was a statistically significant difference (p = 0.0013) in mortality between the out-of-hours (28.2%) and in-hours group (31.9%).

The data shows convincing evidence that patients have a worse outcome when admitted during normal working hours even though in-hours, there is increased access to hospital resources (doctors, nurses, investigations and specialist services) compared to out-of-hours. Interestingly, when extrapolated further, survival worsens particularly in the first few hours of the working day. This corresponds to the time of day where ICU has the fewest number of admissions (Figure 2).

We can only speculate as to the underlying cause of this result and further work will need to be completed in order to understand the causative factors. This will include assessing staff availability to review patients or action management plans and delayed admissions from overnight.

## Conclusions

There is a survival benefit for patients admitted out-of-hours compared to those admitted during in-hours. Further studies need to done in order to ascertain the factors that contribute to this finding.

**Figure 1:**
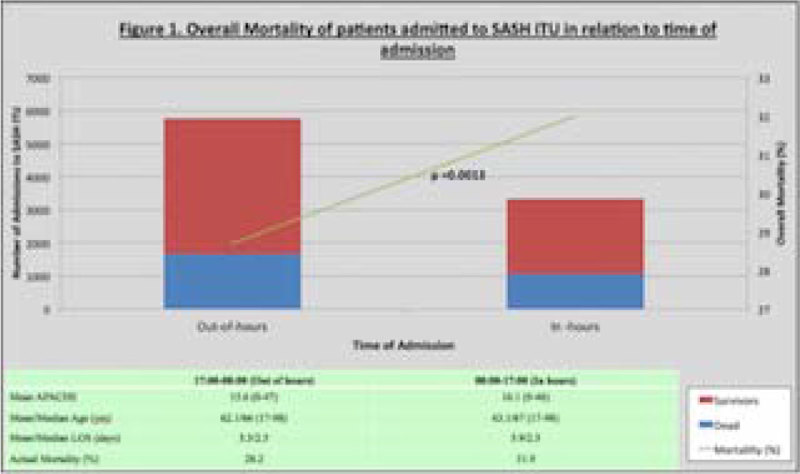
**Overall Mortality of patients admitted to SASH ITU**.
